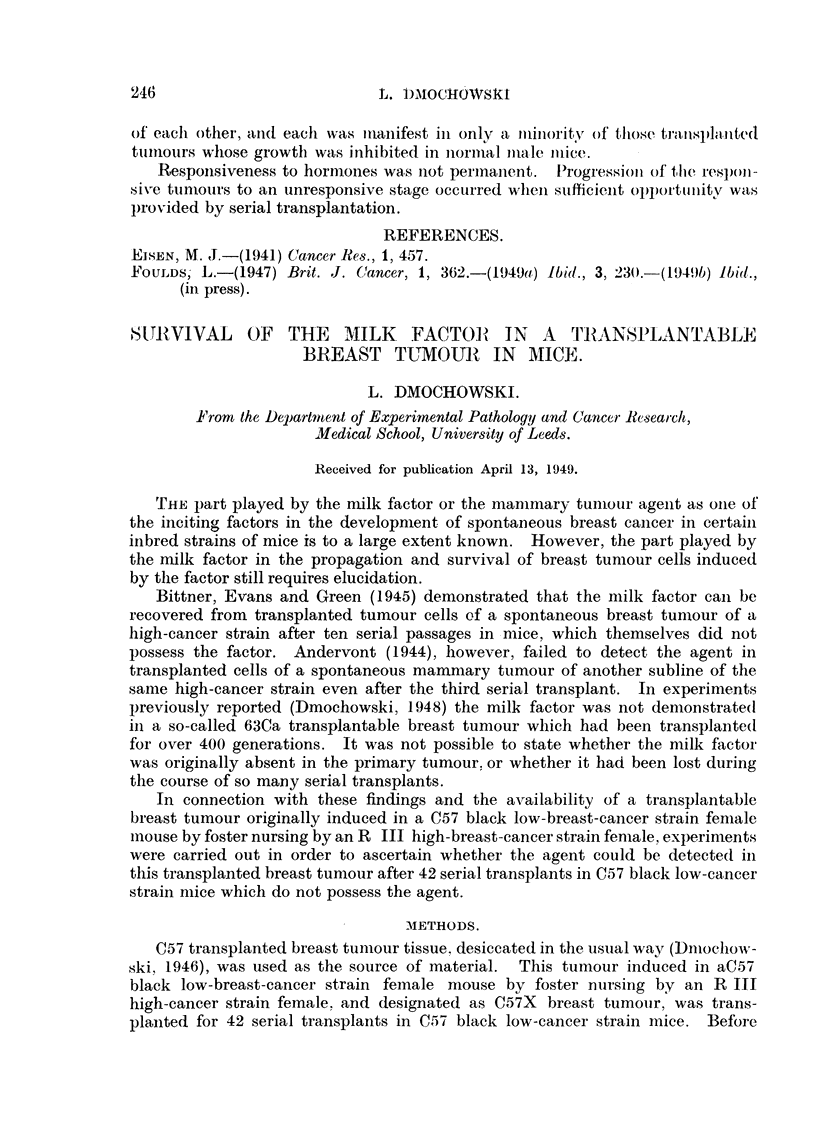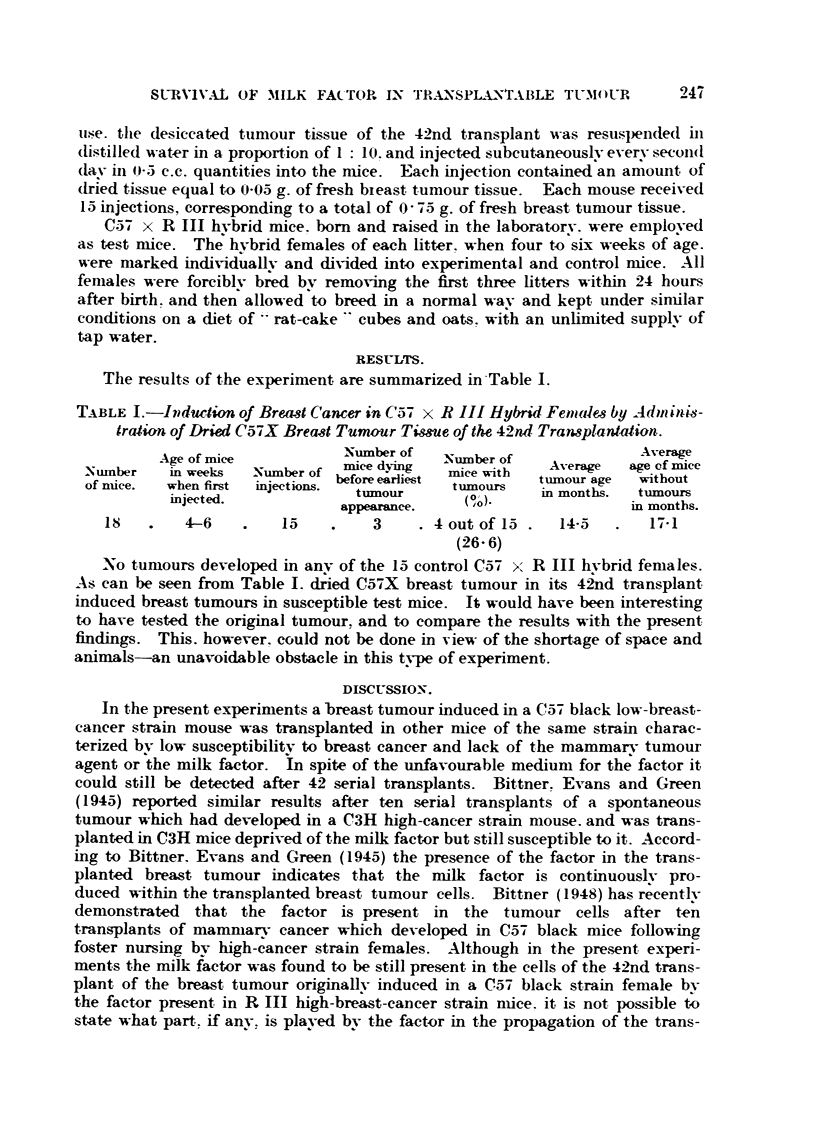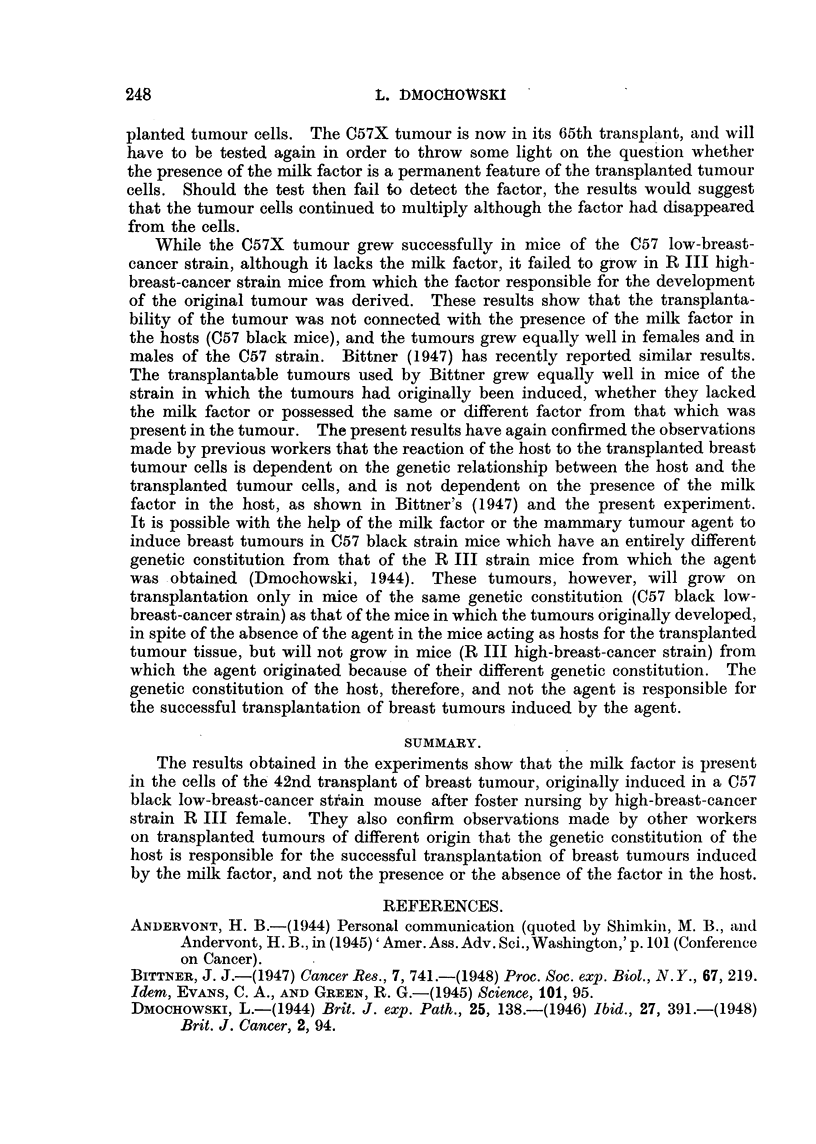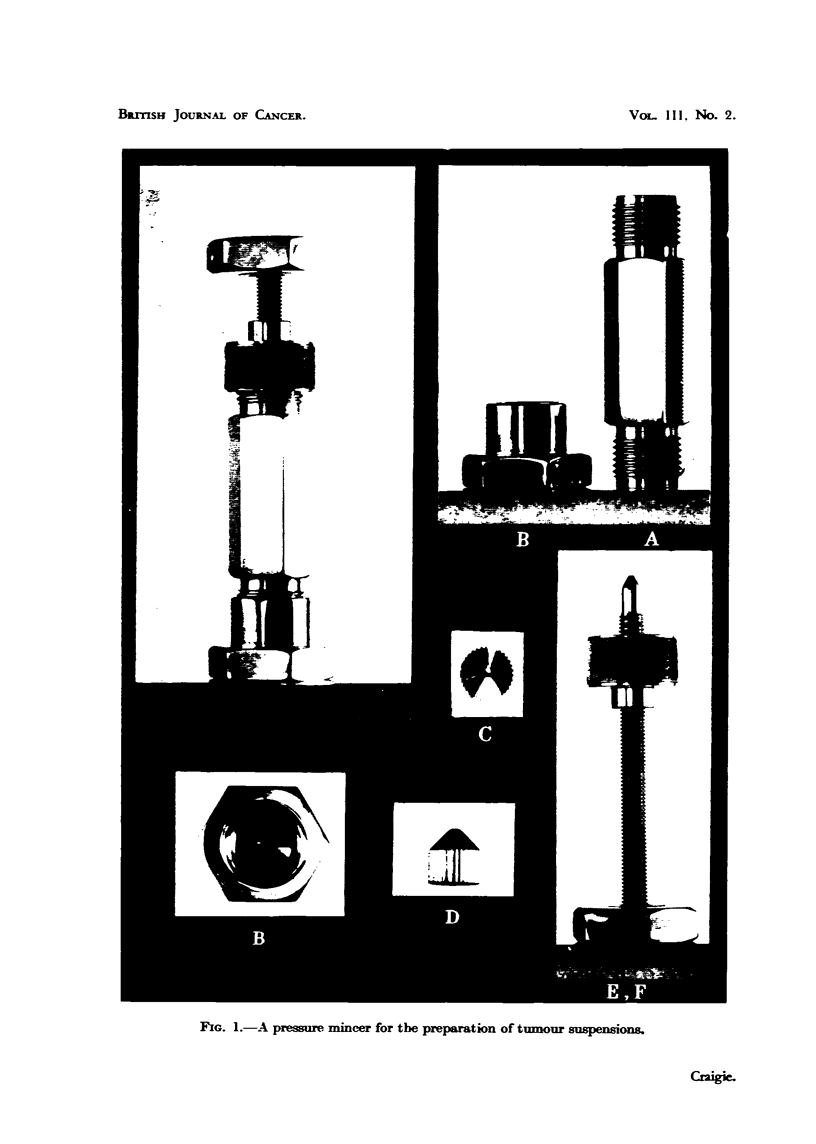# Survival of the Milk Factor in a Transplantable Breast Tumour in Mice

**DOI:** 10.1038/bjc.1949.25

**Published:** 1949-06

**Authors:** L. Dmochowski

## Abstract

**Images:**


### REFERENCES.

EISEN, M. J.—(1941) *Cancer Res.*, **1**, 457.
FOULDS, L.—(1947) *Brit. J. Cancer*, **1**, 362.—(1949*a*) *Ibid.*, **3**, 230.—(1949*b*) *Ibid.*, (in press).

### SURVIVAL OF THE MILK FACTOR IN A TRANSPLANTABLE BREAST TUMOUR IN MICE.

L. DMOCHOWSKI.

*From the Department of Experimental Pathology and Cancer Research, Medical School, University of Leeds.*

Received for publication April 13, 1949.

THE part played by the milk factor or the mammary tumour agent as one of the inciting factors in the development of spontaneous breast cancer in certain inbred strains of mice is to a large extent known. However, the part played by the milk factor in the propagation and survival of breast tumour cells induced by the factor still requires elucidation.

Bittner, Evans and Green (1945) demonstrated that the milk factor can be recovered from transplanted tumour cells of a spontaneous breast tumour of a high-cancer strain after ten serial passages in mice, which themselves did not possess the factor. Andervont (1944), however, failed to detect the agent in transplanted cells of a spontaneous mammary tumour of another subline of the same high-cancer strain even after the third serial transplant. In experiments previously reported (Dmochowski, 1948) the milk factor was not demonstrated in a so-called 63Ca transplantable breast tumour which had been transplanted for over 400 generations. It was not possible to state whether the milk factor was originally absent in the primary tumour, or whether it had been lost during the course of so many serial transplants.

In connection with these findings and the availability of a transplantable breast tumour originally induced in a C57 black low-breast-cancer strain female mouse by foster nursing by an R III high-breast-cancer strain female, experiments were carried out in order to ascertain whether the agent could be detected in this transplanted breast tumour after 42 serial transplants in C57 black low-cancer strain mice which do not possess the agent.

### METHODS.

C57 transplanted breast tumour tissue, desiccated in the usual way (Dmochowski, 1946), was used as the source of material. This tumour induced in aC57 black low-breast-cancer strain female mouse by foster nursing by an R III high-cancer strain female, and designated as C57X breast tumour, was transplanted for 42 serial transplants in C57 black low-cancer strain mice. Before use. the desiccated tumour tissue of the 42nd transplant was resuspended in distilled water in a proportion of 1 : 10, and injected subcutaneously every second day in 0·5 c.c. quantities into the mice. Each injection contained an amount of dried tissue equal to 0·05 g. of fresh breast tumour tissue. Each mouse received 15 injections, corresponding to a total of 0·75 g. of fresh breast tumour tissue.

C57 × R III hybrid mice, born and raised in the laboratory, were employed as test mice. The hybrid females of each litter, when four to six weeks of age, were marked individually and divided into experimental and control mice. All females were forcibly bred by removing the first three litters within 24 hours after birth, and then allowed to breed in a normal way and kept under similar conditions on a diet of "rat-cake" cubes and oats, with an unlimited supply of tap water.

### RESULTS.

The results of the experiment are summarized in Table I.

TABLE I.—*Induction of Breast Cancer in C57 × R III Hybrid Females by Administration of Dried C57X Breast Tumour Tissue of the 42nd Transplantation.*

| Number of mice. | Age of mice in weeks when first injected. | Number of injections. | Number of mice dying before earliest tumour appearance. | Number of mice with tumours (%). | Average tumour age in months. | Average age of mice without tumours in months. |
|---|---|---|---|---|---|---|
| 18 | 4–6 | 15 | 3 | 4 out of 15 (26·6) | 14·5 | 17·1 |

No tumours developed in any of the 15 control C57 × R III hybrid females. As can be seen from Table I, dried C57X breast tumour in its 42nd transplant induced breast tumours in susceptible test mice. It would have been interesting to have tested the original tumour, and to compare the results with the present findings. This, however, could not be done in view of the shortage of space and animals—an unavoidable obstacle in this type of experiment.

### DISCUSSION.

In the present experiments a breast tumour induced in a C57 black low-breast-cancer strain mouse was transplanted in other mice of the same strain characterized by low susceptibility to breast cancer and lack of the mammary tumour agent or the milk factor. In spite of the unfavourable medium for the factor it could still be detected after 42 serial transplants. Bittner, Evans and Green (1945) reported similar results after ten serial transplants of a spontaneous tumour which had developed in a C3H high-cancer strain mouse, and was transplanted in C3H mice deprived of the milk factor but still susceptible to it. According to Bittner, Evans and Green (1945) the presence of the factor in the transplanted breast tumour indicates that the milk factor is continuously produced within the transplanted breast tumour cells. Bittner (1948) has recently demonstrated that the factor is present in the tumour cells after ten transplants of mammary cancer which developed in C57 black mice following foster nursing by high-cancer strain females. Although in the present experiments the milk factor was found to be still present in the cells of the 42nd transplant of the breast tumour originally induced in a C57 black strain female by the factor present in R III high-breast-cancer strain mice, it is not possible to state what part, if any, is played by the factor in the propagation of the transplanted tumour cells. The C57X tumour is now in its 65th transplant, and will have to be tested again in order to throw some light on the question whether the presence of the milk factor is a permanent feature of the transplanted tumour cells. Should the test then fail to detect the factor, the results would suggest that the tumour cells continued to multiply although the factor had disappeared from the cells.

While the C57X tumour grew successfully in mice of the C57 low-breast-cancer strain, although it lacks the milk factor, it failed to grow in R III high-breast-cancer strain mice from which the factor responsible for the development of the original tumour was derived. These results show that the transplantability of the tumour was not connected with the presence of the milk factor in the hosts (C57 black mice), and the tumours grew equally well in females and in males of the C57 strain. Bittner (1947) has recently reported similar results. The transplantable tumours used by Bittner grew equally well in mice of the strain in which the tumours had originally been induced, whether they lacked the milk factor or possessed the same or different factor from that which was present in the tumour. The present results have again confirmed the observations made by previous workers that the reaction of the host to the transplanted breast tumour cells is dependent on the genetic relationship between the host and the transplanted tumour cells, and is not dependent on the presence of the milk factor in the host, as shown in Bittner's (1947) and the present experiment. It is possible with the help of the milk factor or the mammary tumour agent to induce breast tumours in C57 black strain mice which have an entirely different genetic constitution from that of the R III strain mice from which the agent was obtained (Dmochowski, 1944). These tumours, however, will grow on transplantation only in mice of the same genetic constitution (C57 black low-breast-cancer strain) as that of the mice in which the tumours originally developed, in spite of the absence of the agent in the mice acting as hosts for the transplanted tumour tissue, but will not grow in mice (R III high-breast-cancer strain) from which the agent originated because of their different genetic constitution. The genetic constitution of the host, therefore, and not the agent is responsible for the successful transplantation of breast tumours induced by the agent.

### SUMMARY.

The results obtained in the experiments show that the milk factor is present in the cells of the 42nd transplant of breast tumour, originally induced in a C57 black low-breast-cancer strain mouse after foster nursing by high-breast-cancer strain R III female. They also confirm observations made by other workers on transplanted tumours of different origin that the genetic constitution of the host is responsible for the successful transplantation of breast tumours induced by the milk factor, and not the presence or the absence of the factor in the host.

### REFERENCES.

ANDERVONT, H. B.—(1944) Personal communication (quoted by Shimkin, M. B., and Andervont, H. B., in (1945) 'Amer. Ass. Adv. Sci., Washington,' p. 101 (Conference on Cancer).

BITTNER, J. J.—(1947) *Cancer Res.*, **7**, 741.—(1948) *Proc. Soc. exp. Biol., N.Y.*, **67**, 219.

*Idem*, EVANS, C. A., AND GREEN, R. G.—(1945) *Science*, **101**, 95.

DMOCHOWSKI, L.—(1944) *Brit. J. exp. Path.*, **25**, 138.—(1946) *Ibid.*, **27**, 391.—(1948) *Brit. J. Cancer*, **2**, 94.

FIG. 1.—A pressure mincer for the preparation of tumour suspensions.